# New onset ANCA-associated vasculitis in an adolescent during an acute COVID-19 infection: a case report

**DOI:** 10.1186/s12887-021-02812-y

**Published:** 2021-08-05

**Authors:** Daniel D. Reiff, Chloe G. Meyer, Brittany Marlin, Melissa L. Mannion

**Affiliations:** 1 Children’s of Alabama, Division of Rheumatology, Department of Pediatrics, University of Alabama at Birmingham, 1600 7th Ave. S., CPPN #G10, Birmingham, AL 35233-1711 USA; 2 UAB Pediatric Residency Program, University of Alabama at Birmingham, Birmingham, AL USA; 3 Division of Pediatric Hospital Medicine, Department of Pediatrics, University of Alabama at Birmingham, Birmingham, AL USA

**Keywords:** COVID-19, ANCA associated vasculitis, Autoimmunity, Pediatrics, Case report

## Abstract

**Background:**

SARS-CoV-2 has been found to be exquisitely adept at triggering autoimmunity and multiple new onset autoimmune diseases have been described as a post-infectious complication of COVID-19 infection in the adult population. Less has been described in the pediatric population, as infections are more likely to be asymptomatic and less severe. This case reports a previously healthy adolescent patient with new onset antineutrophil cytoplasmic autoantibody-associated vasculitis (AAV) diagnosed in the setting of acute COVID-19 infection.

**Case presentation:**

A previously healthy adolescent male was diagnosed with COVID-19 pneumonia after presenting with infectious symptoms of fever, cough, congestion, and shortness of breath. After worsening of disease, he was found to have pulmonary nodules, atypical for COVID-19. Further imaging and laboratory workup showed elevated inflammatory markers, negative infectious testing, and positive antineutrophil cytoplasmic antibodies (ANCA) diagnostic for AAV. He was treated with pulse dose steroids followed by a prolonged taper and rituximab. Symptoms resolved and laboratory abnormalities improved over time. At six-month follow-up, lesions were much improved, laboratory markers were within normal limits, and patient remained asymptomatic off medications.

**Conclusions:**

This case is one of the first in the pediatric population to describe new onset AAV presenting with an acute, symptomatic COVID-19 infection. There is increasing evidence for COVID-19 induced autoimmunity in the pediatric population and pediatric care providers should be on high alert for new onset autoimmune disease in children afflicted by COVID-19.

## Background

Coronavirus disease 2019 (COVID-19) caused by severe acute respiratory syndrome coronavirus 2 (SARS-CoV-2) has caused a high degree of morbidity and mortality since its appearance in late 2019. Fortunately, COVID-19 has been relatively less severe in the pediatric and adolescent population, with children more likely than adults to be asymptomatic or have mild infectious symptoms [1, 2]. However, multi-system inflammatory syndrome in children (MIS-C) has since emerged as a post-infectious cause of severe illness in the pediatric population, thought to be due to a vasculitic and autoimmune pathogenesis [3]. In general, SARS-CoV-2 has been hypothesized to be an exquisitely adept trigger of autoimmunity, with multiple reports of secondary autoimmune diseases after initial COVID-19 infection in the adult population [4]. Less has been published on COVID-19 triggered autoimmune disease beyond MIS-C in the pediatric and adolescent population. Herein, we describe a case of new onset antineutrophil cytoplasmic autoantibody-associated vasculitis (AAV) in the setting of acute COVID-19 infection in an adolescent patient, to bring increased awareness to autoimmune sequelae of COVID-19.

## Case presentation

**Fig. 1:**
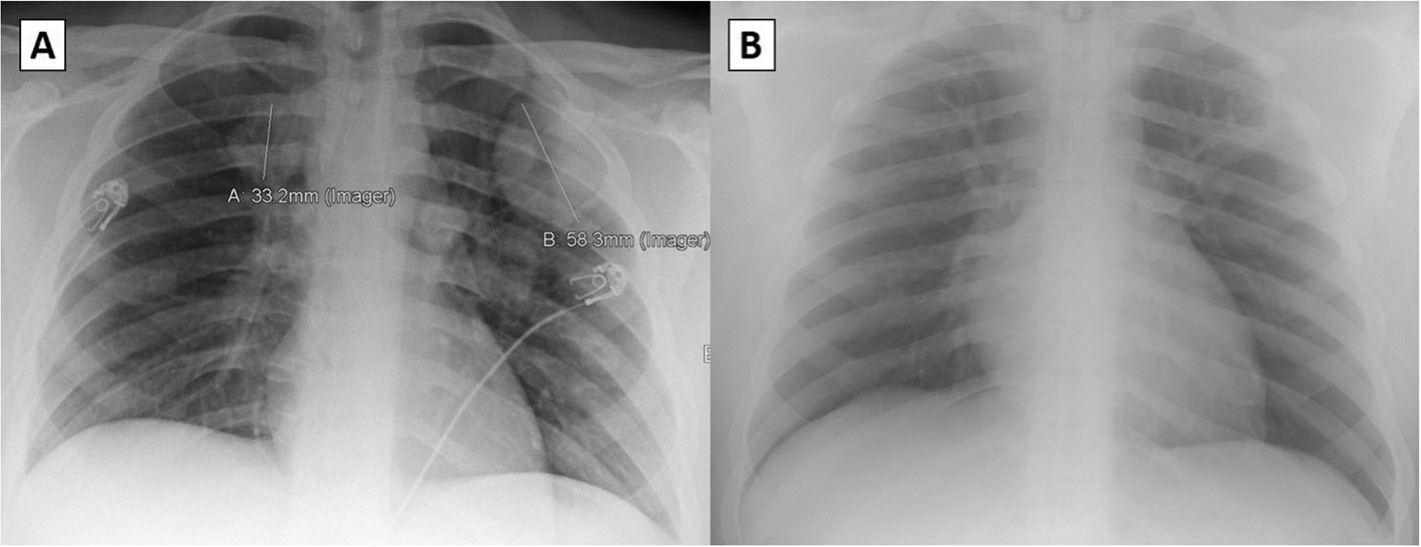
Chest x-ray findings at presentation (**A**) and 3-month follow-up (**B**)

**Fig. 2:**
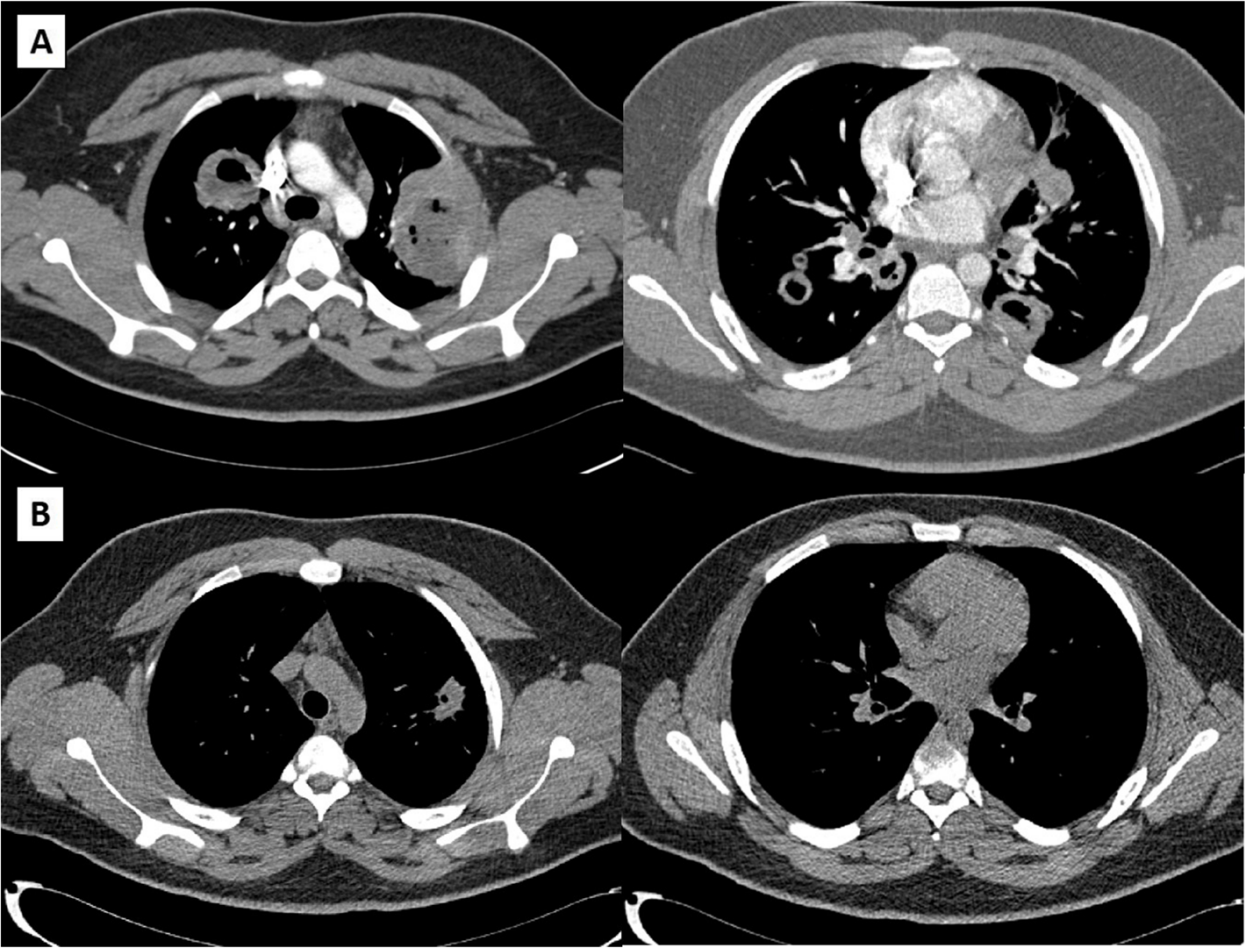
Chest CT findings at presentation (**A**) and 6-month follow-up (**B**)

A 17-year-old, previously healthy male developed fevers to a maximum temperature of 102 °F, drenching night sweats, cough, nasal congestion, and chest tightness. One week prior to the onset of illness, the patient and his family were exposed to a COVID-19 positive sick contact, so at the onset of symptoms, our patient was tested and returned positive for COVID-19 via nasal polymerase chain reaction (PCR). For the next seven days, these infectious symptoms continued including a five-pound weight loss during this time. On day seven of illness, the patient complained of acute worsening of shortness of breath, lightheadedness, two episodes of hemoptysis, and was noted to have an oxygen saturation of 86% on a home oxygen monitor. He was then brought to a local community hospital for evaluation on day seven of illness for evaluation. On arrival, patient was febrile to 101.2 °F, but otherwise had reassuring respiratory rate, regular heart rate, normal lung exam, and oxygen saturation of 94% on room air. Repeat COVID-19 PCR again was positive. On a routine chest x-ray, he was found to have a 5 cm left upper lobe mass and a 3 cm right paratracheal mass (Fig. 1). Concurrent lab findings noted a normal complete blood count, electrolytes, kidney function testing, and liver function tests. To better delineate chest x-ray findings, patient underwent a chest computed tomography (CT) which showed multiple bilateral cavitary lung lesions, largest within the left upper lobe measuring 6.5 cm in diameter (Fig. 2). With these findings, patient was transferred to our tertiary care pediatric hospital for further management.

The patient arrived to our institution on day eight of illness and was noted to be afebrile, continued to have normal oxygen saturations on room air, and his physical exam was largely unremarkable with good aeration in all lung fields and no crackles or focality on auscultation, normal neurologic status, no abnormal skin findings, and reassuring cardiac exam. He was placed on airborne precautions due to previous positive COVID-19 PCR, and COVID-19 PCR screening again returned positive on admission. Initial laboratory testing showed depressed white blood cell count (WBC) at 2.91 × 10^3^/uL, hemoglobin normal at 14.2 g/dL, and a normal platelet count of 369 × 10^3^/uL. His kidney function was normal with creatinine of 0.74 mg/dL and patient had normal electrolytes with only slight elevation in alanine transaminase level (ALT) to 35.8 U/L. Inflammatory markers were elevated with C - reactive protein (CRP) found to be 4.52 mg/dL and an erythrocyte sedimentation rate (ESR) of 55 mm/hr. Urinalysis was negative for protein, WBCs, and blood. Due to broad infectious differential, patient was placed on vancomycin and ceftriaxone, and completed a 48-h rule-out. From an infectious standpoint, patient had testing return negative for HIV, tuberculosis, aspergillus, Histoplasma, Cryptococcus, and blood cultures remained negative throughout his hospital course, from illness day eight through day eleven. SARS-CoV-2 antibodies were not tested on presentation. With embolic phenomena on the differential, patient had a normal echocardiogram performed and upper/lower extremity venous Doppler ultrasounds were negative for deep venous thrombosis. Additionally, with MIS-C on the differential, ferritin, d-dimer, fibrinogen, and troponin levels were obtained. Ferritin, d-dimer, and fibrinogen levels were slightly elevated consistent with an inflammatory process, but with normal troponin levels, normal echocardiogram, lack of hypotension, and downtrend of fever curve without treatment, concern for MIS-C decreased substantially. With a largely reassuring work-up and improvement in fever curve, the patient was discharged from his hospitalization on day eleven of illness.

After discharge, sarcoidosis testing returned negative with normal angiotensin converting enzyme levels and lysozyme levels. On day thirteen of illness, cytoplasmic antineutrophil cytoplasmic autoantibody (c-ANCA) testing returned elevated with a titer > 1:640 and a proteinase 3 antibody (PR3) level of 251.9 (normal < 1.0). With concern for the possibility of AAV, the patient was set up for a percutaneous lung biopsy that was performed on illness day twenty-one. Between discharge on day eleven and day twenty-one, our patient continued to have cough, intermittent hemoptysis, and daily fever. Additionally, patient’s first negative SARS-CoV-2 PCR occurred on illness day 21 prior to his lung biopsy. Biopsy of the largest lesion was negative for any infectious agents on culture, but was notable for mixed perivascular inflammation and necrotic debris. So with highly positive c-ANCA and PR3 antibodies, and CT findings of pulmonary nodules in the setting of negative infectious testing, a diagnosis of AAV was made based, as patient did not meet all required criteria for granulomatosis with polyangiitis (GPA) [5] [6]. For a complete AAV workup, at the time of lung biopsy, a sinus CT was performed which returned normal and repeat kidney function was reassuring with normal creatinine and normal urine protein/creatinine ratio. As treatment for pulmonary-limited AAV, the patient had two doses of pulse-dose methylprednisolone (1 g on illness days twenty-one and twenty-two) and received two doses of Rituximab (1 g on illness days twenty-one and thirty-five) for definitive treatment. Over the next 3 months, the patient underwent a prednisone wean (initial dose 60 mg, 0.6 mg/kg/day) with regular laboratory checks and outpatient clinic appointments. Shortly after pulse dose steroids and Rituximab, he had resolution of his fevers and constitutional symptoms, and during the steroid wean he continued to be asymptomatic with steady reduction in c-ANCA titers, PR3 levels, and inflammatory markers (Table 1). On discontinuation of steroids at the three-month mark, a repeat chest x-ray was performed which showed residual small cavitary lesions in the right and left upper lung markedly improved from the previous x-ray (Fig. 1). At his 6-month follow-up, repeat chest CT showed significant improvement, with prior noted multiple cavitary nodules either resolved or markedly reduced in size (Fig. 2). With the improvement in imaging, continued asymptomatic status, and stable labs without evidence of ongoing inflammation, the patient will continue to be followed off medication with regular screenings for return of symptoms or laboratory evidence of inflammation.

**Table 1:** Laboratory Findings Throughout Illness

	Presentation	1 month^a^	2 months^b^	3 months^c^	6 months^d^
White blood cell count (× 10^3/uL)	2.91	16.16	12.82	6.05	4.93
Hemoglobin (g/dL)	14.2	13.9	15	15.4	16.5
Platelet Count (×10^3/uL)	369	415	420	354	319
Erythrocyte Sedimentation Rate (mm/hr)	88	14	9	6	3
C Reactive Protein (mg/dL)	13.18	0.23	0.34	0.11	0.1
c-ANCA titer	> 1:640	–	1:160	1:80	1:80
Proteinase 3 Ab (AI)	251.9	–	25.1	20.5	7.5

^a^0.6 mg/kg/day prednisone, s/p Rituximab

^b^0.2 mg/kg/day prednisone

^c^0.05 mg/kg/day prednisone

^d^Off all medication

## Discussion and conclusions

This case is one of the first to report autoimmune ANCA-associated vasculitis in the setting of an acute pediatric COVID-19 infection. One case of pediatric, post-infectious anti-MPO (myeloperoxidase) vasculitis has been previously reported in the literature after a patient with diffuse alveolar hemorrhage was found to be positive for SARS-CoV-2 IgG antibodies [7]. And while MIS-C is a widely reported post-infectious manifestation of COVID-19 in the pediatric population, little has been reported about onset of autoimmune disease or vasculitis in the acute phase of COVID-19 in children or adolescents. What has been reported in the pediatric literature to date consists mainly of case reports or case series, including limited examples of chilblains as a cutaneous vasculitic COVID-19 manifestation [8] and autoimmune neurologic sequelae [9, 10]. In contrast, there is a wider variety and larger amount of adult acute COVID-related autoimmune diseases noted in the literature. Autoimmune hemolytic anemia [11–13], Guillain-Barré syndrome [14], immune thrombocytopenic purpura [15], and ANCA-associated vasculitis with glomerulonephritis [16] are just a few autoimmune diseases that have been reported in adults suffering from acute COVID-19.

It has long been hypothesized that viral triggers are extremely important in the pathogenesis of autoimmune diseases [17], however it is still uncertain exactly how SARS-CoV-2 brings about this propensity for autoimmunity. One current theory includes general hyper-stimulation of the immune system, leading to elevated levels of pro-inflammatory cytokines and cytokine storm syndrome [18]. Another hypothesizes the loss of immune tolerance to self-antigens due to transient immunosuppression, leading to the development of autoantibodies and failure to properly recognize self-antigens [19]. Overall, more investigation is needed into the pathogenesis of COVID-related autoimmunity.

It is somewhat difficult to completely rule-out the possibility of our patient having asymptomatic AAV pulmonary nodules prior to his acute COVID-19 infection that was brought to light by the screening x-ray for his infectious symptoms. However, given the rarity of AAV, it is reasonable to believe that this case represents a true example of COVID induced autoimmunity, especially with ANCA positivity reported in previous cases of active COVID-19 [4, 20]. Additionally, when examining large population studies of MIS-C and pediatric COVID-19, we have found no mention of cavitary lesions as a sequelae of either disease process. The respiratory symptoms and imaging findings in MIS-C seem to be largely secondary to vascular leak and cardiac abnormalities including ARDS, pulmonary edema, and pleural effusions [21–23], whereas the findings in acute COVID-19 are more of a typical pneumonic picture with ground-glass opacities and pulmonic infiltrates [24]. Overall, this case makes it clear that providers should be on high alert for new onset autoimmune diseases in the pediatric population in the upcoming years, both as a post-infectious complication of SARS-CoV-2 and in the setting of acute COVID-19 infection.

## Data Availability

The datasets used and/or analysed during the current study are available from the corresponding author on reasonable request.

## Abbreviations

COVID-19: Coronavirus disease 2019
SARS-CoV-2: severe acute respiratory syndrome coronavirus 2
MIS-C: multi-system inflammatory syndrome in children
AAV: ANCA-associated vasculitis
GPA: Granulomatosis with polyangiitis
PCR: polymerase chain reaction
CT: computed tomography
WBC: white blood cell count
ALT: alanine transaminase level
CRP: C-reactive protein
ESR: erythrocyte sedimentation rate
ANCA: antineutrophil cytoplasmic antibody
PR3: proteinase 3 antibody
MPO: myeloperoxidase
